# Optimizing whole-genomic prediction for autotetraploid blueberry breeding

**DOI:** 10.1038/s41437-020-00357-x

**Published:** 2020-10-19

**Authors:** Ivone de Bem Oliveira, Rodrigo Rampazo Amadeu, Luis Felipe Ventorim Ferrão, Patricio R. Muñoz

**Affiliations:** Blueberry Breeding and Genomics Lab, Horticultural Sciences Department, University of Florida, Gainesville, FL 32611 USA

## Abstract

Blueberry (*Vaccinium* spp.) is an important autopolyploid crop with significant benefits for human health. Apart from its genetic complexity, the feasibility of genomic prediction has been proven for blueberry, enabling a reduction in the breeding cycle time and increasing genetic gain. However, as for other polyploid crops, sequencing costs still hinder the implementation of genome-based breeding methods for blueberry. This motivated us to evaluate the effect of training population sizes and composition, as well as the impact of marker density and sequencing depth on phenotype prediction for the species. For this, data from a large real breeding population of 1804 individuals were used. Genotypic data from 86,930 markers and three traits with different genetic architecture (fruit firmness, fruit weight, and total yield) were evaluated. Herein, we suggested that marker density, sequencing depth, and training population size can be substantially reduced with no significant impact on model accuracy. Our results can help guide decisions toward resource allocation (e.g., genotyping and phenotyping) in order to maximize prediction accuracy. These findings have the potential to allow for a faster and more accurate release of varieties with a substantial reduction of resources for the application of genomic prediction in blueberry. We anticipate that the benefits and pipeline described in our study can be applied to optimize genomic prediction for other diploid and polyploid species.

## Introduction

Genomic prediction, originally proposed for animal breeding (Meuwissen et al. 2001), involves the use of genomic information to predict the genetic merit of untested genotypes. This is built upon the premise of existence of linkage disequilibrium between causal polymorphisms and the molecular markers used in the analysis (Meuwissen et al. 2001; Zhang et al. 2011; Daetwyler et al. 2013; de los Campos et al. 2013). The predictive model is derived from an extensively phenotyped and genotyped reference population, in a so-called training step. After validation, the model is used to predict the genomic breeding value of candidates in a selection set. Therefore, marker effects estimated in the training population should be predictable in the selection population when linkage disequilibrium is maintained across populations (Asoro et al. 2011). This methodology has revolutionized plant breeding, allowing breeders to perform accurate selections of superior genotypes in early stages, skipping breeding phases, reducing costs associated with field trials and phenotyping, and increasing the rate of genetic gain per unit of time (Crossa et al. 2017). Despite its importance, implementing genomic prediction in breeding programs is challenging since it is costly (Spindel et al. 2015; Sverrisdóttir et al. 2017; Norman et al. 2018).

Theory suggests the use of high marker densities and a large number of individuals in the training population to improve model accuracy (Meuwissen et al. 2001). By increasing marker density and distribution, one increases the probability of capturing the association between markers and causal loci, while increasing training population size helps to avoid ascertainment bias, improving the estimation of marker effects (Meuwissen et al. 2001; de los Campos et al. 2013; Spindel et al. 2015). Not only size, but also the genetic composition of the training population is critical, as only the genetic variation that is present in it will be used to build the prediction model. Thus, the training population should contain the most informative set of individuals (Lorenz and Smith 2015), and be somewhat representative of the population to which the model will be applied (Habier et al. 2007, 2010). Kinship and population structure can also be taken into account when developing prediction models (de los Campos and Sorensen 2014).

Even though predictive performance tends to improve with the increase of training population size and marker density, a plateau is normally reached (Cericola et al. 2017; Norman et al. 2018). The expansion in training population size would significantly increase the model development costs, since in this set all individuals should be genotyped and phenotyped. Therefore, optimized values can and should be established for marker number, and for training population size and composition. Such optimization can guide the construction of genomic prediction models with high accuracy and a low budget (Isidro et al. 2015; Spindel et al. 2015; Cericola et al. 2017, 2018; Abed et al. 2018).

Another factor that plays a key role in model development costs when using next generation sequencing is sequencing depth (i.e., the number of reads sequenced for a given site in the genome), which is extremely important in the polyploid context. Polyploidy is a common event in plants, as about 70% of all angiosperms and 95% of all pteridophytes underwent polyploidization during their evolution (Soltis and Soltis 1999). These species present more than two homo(e)logous copies of each chromosome, where each one of them can carry different alleles. Polyploids are of great importance in agriculture, representing numerous species classified as world’s staple crops (e.g., wheat, rye, oat, potatoes, yams, taro, and sugarcane). Breeding polyploids is challenging compared to diploid species, since they can present genotypes with higher allele dosage (i.e., the number of times that an allele is present in a specific locus) resulting in a larger number of genotypic classes when compared to diploid species. This leads to the possibility of higher orders of allele interaction (see Gallais 2003). In addition, polyploids commonly present high heterozygosity and possibility of multivalent pairing (see Qu et al. 1998 for details). All these factors add complexity to the use of molecular data information, and therefore, to the application of genome-based breeding methods.

The use of low sequencing depth in polyploids can result in a sampling of a biased subset of alleles which might misrepresent the real genotype of the locus (Caruana et al. 2019). This can ultimately affect genomic prediction performance. Moreover, next generation sequencing continues to suffer from high error rates, which can generate further problems with the misclassification of genotypic classes. This bias in genotyping can affect the results in association studies (Grandke et al. 2016). To circumvent this bias, it has been proposed to sequence polyploid species at higher sequencing depth. For autotetraploid species, such as blueberry (*Vaccinium* spp.), sequencing depths of 50X–80X have been recommended to achieve confidence in the allele dosage estimation process (Uitdewilligen et al. 2013; Bastien et al. 2018). Even though there is a direct and positive association between the increase in sequencing depth and the quality of the called genotypes, this can also cause an increase in genotyping costs (Gorjanc et al. 2015, 2017; Caruana et al. 2019). For diploid animal breeding, studies have proven that a sequencing depth of 1X is effective to obtain high levels of accuracy in large breeding populations (Gorjanc et al. 2015, 2017). This reduction in sequencing depth could significantly decrease genotyping costs. However, to our knowledge, no autopolyploid study has yet investigated the influence of sequencing depth on genomic prediction. Herein, by using a large dataset sequenced at high coverage, we propose to investigate the impact of sequencing depth on prediction for three fruit quality traits in blueberry—with different genetic architectures.

Genome-based breeding methodologies are starting to be applied to blueberry breeding (e.g., Ferrão et al. 2018; Amadeu et al. 2019; de Bem Oliveira et al. 2019). The feasibility of genomic prediction has been proven for blueberry, and promising results are expected. Implementing this methodology to the selection process would lead to an average increase of 86% for expected genetic gain and reduce breeding cycle time from 12 to 6 years (de Bem Oliveira et al. 2019). However, the high investment required for genotyping is still one of the major challenges to the practical application of genomic prediction (Sverrisdóttir et al. 2017), and no study has yet been performed to investigate how this process could be optimized for blueberry. Therefore, the objective of this research was to evaluate the effect of marker density, sequencing depth, and training population size and composition in order to generate a cost-effective application of genomic prediction. We anticipate that our findings can also facilitate the task of implementing genomic selection beyond blueberry.

## Material and methods

### Population and phenotyping

The blueberry genotypes included in this study comprise a representative population of the University of Florida Blueberry Breeding Program (as described in Cellon et al. 2018; Ferrão et al. 2018; de Bem Oliveira et al. 2019). In summary, this population encompassed 1804 genotypes originated from 117 biparental-designed crosses of 146 parents. Genotypes were evaluated in two production seasons (2014 and 2015). To maximize divergence on the genetic control and heritability of the traits, three of the eight phenotypes evaluated in previous studies were investigated: (i) fruit weight (g), (ii) fruit firmness (g mm^−1^ of compression force), and (iii) total yield (1–5 scale). Fruit weight and fruit firmness measurements were obtained from five randomly sampled fully mature berries. To measure weight, an analytical scale was used (CP2202S, Sartorious Corp., Bohemia, NY). Firmness values were obtained with the FirmTech II firmness tester (BioWorks Inc., Wamego, KS). Yield was evaluated using a 1 (low) to 5 (high) rating scale based on visual assessment.

Least square means (LSMeans) were obtained for all genotypes using a single trait analysis. The linear model considered genotype and year as fixed effects (as implemented by Amadeu et al. 2019). This linear model was fitted in R with the lm function within the stats package (R Development Core Team 2019). Adjusted means (i.e., LSMeans) were extracted using the lsMeans package (Lenth 2016). Subsequently, these corrected phenotypes were used as an input for the genomic prediction analyses.

### Genotypic data

Genotypes were obtained using capture-seq and processed as described by Benevenuto et al. (2019). In summary, 15,663 120-mer biotinylated probes designed based on the 2013 blueberry draft genome sequence were used (Bian et al. 2014; Gupta et al. 2015). Probes were aligned to a high-quality draft genome (Colle et al. 2019), using BLAST (Altschul et al. 1990). Probes that aligned uniquely and within homologous groups were selected, resulting in 9390 probes used during single nucleotide polymorphisms (SNP) calling steps. A total of 276,212 SNPs were identified using FreeBayes v.1.0.1 (Garrison and Marth 2012), considering the tetraploid option.

### Marker data and filtering

Only SNPs that met the following criteria were retained for further analysis: (i) minimum mapping quality score of 20; (ii) minimum SNP phred quality score of 10; (iii) biallelic markers; (iv) maximum genotype and marker missing data of 0.2; and (v) minor population allele frequency of 0.05. In addition, markers were kept when presenting average sequencing depth per site across all individuals of 60X. To avoid the use of imputation methods, it was required that all data points presented a minimum sequencing depth of 2X. A total of 87,628 SNPs were obtained after filtering, and only SNPs on the scaffolds associated with blueberry chromosomes (Table S1) were kept, totalizing 86,930 SNPs, which were used in the genomic prediction analysis (presenting average sequencing depth per sample of 76X). The minimum limit of sequencing depth = 60X was chosen to improve the analysis, using only markers with high-quality scores. Sequencing read counts per allele and individual were extracted from the variant call file using the vcfR package (Knaus and Grundwald 2017).

Continuous genotypes were used for all tests following this formula: #a/(#A + #a), where (#a) and (#A) refer to the sequencing depth for the alternative and the reference allele, respectively, as described by de Bem Oliveira et al. (2019).

### Marker density

To evaluate the effect of marker density on phenotype prediction, we obtained nine scenarios of marker filters: 500, 1000 (1k), 2000 (2k), 3000 (3k), 5000 (5k), 10,000 (10k), 20,000 (20k), 40,000 (40k), and 60,000 (60k) markers. Results obtained with these filters were compared with results obtained for the complete set of markers 86,930 (86k). An equal number of markers was sampled from each chromosome. Samplings were independently performed five times for each scenario. In order to avoid eventual bias associated with marker position, a cumulative approach was applied, e.g., the first set of 1k markers was also included into the first set of 2k markers, which was included into the 3k set and so on. Principal component analyses were performed using the R package adegenet v. 1.3-1 (Jombart and Ahmed 2011), in order to obtain the percentage of variance explained in each relationship matrix.

### Sequencing depth

To evaluate the effect of sequencing depth on phenotype prediction, six scenarios were tested. First, as a benchmark, we considered the original number of markers with average sequencing depth of 60X. From that, five new sequencing depth scenarios were sampled and evaluated (i.e., average sequencing depth = 2X, 6X, 12X, 24X, and 48X). To obtain the realized sequencing depth for each of the new scenarios, we assumed a Poisson distribution with the mean corresponding to each sequencing depth scenario. Therefore, for each scenario, the total number of sequence reads (*n*_*ij*_) for the locus *i* of the genotype *j* was obtained assuming *n*_*ij*_ ~ Poisson(sequencing depth), as described by Gorjanc et al. (2015). A minimum sequencing depth of two was established. Five distributions were independently obtained for each sequencing depth scenario. All distributions considered the same marker positions present on the original set (i.e., mean sequencing depth of 60X).

### Probe density

Capture-seq is a genotyping-by-sequencing methodology that uses customizable targeted hybridization technology. To this end, probes complementary to target sequences are designed to cover specific regions on the genome, simplifying the sequencing process. Therefore, the number of probes impacts the number of SNPs and the costs associated with genotyping. Here, we tested the effects of probe density (nprobe) on phenotype prediction by applying seven filters, assuming values between 50 and 5000 probes (i.e., nprobe = 50, 100, 500, 1000, 2000, 3000, 5000). For sampling, a fixed distance between probes was set, and chromosome information was considered (Table S1). To assure random selection of probes and to perform five random samplings for each filter, five random start points were set for each filter. In order to evaluate the combined effect of probe density and sequencing depth on phenotype prediction, the probe analysis was conducted under all five sequencing depth scenarios (2X, 6X, 12X, 24X, 48X, and 60X).

### Training population size and composition

To define how to best create the training population, two approaches were considered: (i) random sampling and (ii) sampling considering family information. As with the number of markers, a cumulative approach was adopted for sampling. For the random scenario, samplings comprising 120, 240, 480, 960, and 1560 individuals were used to create the training populations.

For the scenario considering family information, filters were applied considering a cumulative increase in the number of individuals sampled per family. Only data from families with ten individuals or more were used in these analyses, for a total of 103 families or 1706 genotypes. Training populations tested contained 1, 3, 6, 9, 12, and 15 individuals per family.

In order to understand the interaction between training population size/composition and sequencing depth, all analyses were performed considering four of the sequencing depth scenarios previously described (i.e., 6X, 12X, 24X, and 60X). These scenarios were chosen considering results obtained for the sequencing depth analysis in this study.

### Genomic prediction models

Models were implemented considering the G-BLUP methodology (VanRaden 2008), assuming the following mixed linear model: ${\boldsymbol{y}} = \boldsymbol{\mu} + {\boldsymbol{Xg}} + \boldsymbol{\epsilon}$, where ***y*** is a vector of adjusted phenotypic values, ***X*** is the incidence matrix linking observation in the vector ***y*** to their respective genotype effects in the vector ***g***. Normality was assumed for the additive and residual effects, where ${\boldsymbol{g}} \sim {\mathrm{MVN}}\left( {0,{\boldsymbol{G}}\sigma _a^2} \right),$ and the residual variance $\boldsymbol{\epsilon} \sim {\mathrm{MVN}}(0,{\boldsymbol{I}}\sigma _e^2)$. Genetic covariance, ***G***, was estimated using the *ratio* option in the AGHmatrix R package (Amadeu et al. 2016) as: ${\boldsymbol{G}} = \frac{{{\boldsymbol{ZZ}}^\prime }}{h}$, where the marker matrix ***M*** comprises the ratio values, ***Z*** is the mean-centered ***M***, and *h* is a scale factor, where $h = \mathop {\sum }\nolimits_{i = 0}^m s_i^2$ and $s_i^2$ is the variance of the vector *z*_*i*_ (centered marker vector) (Ashraf et al. 2016; de Bem Oliveira et al. 2019). For the residual, ***I*** was an identity matrix. MVN denotes the *n*-dimensional multivariate normal distribution.

For each combination of trait and scenario, models were individually fit using the R package BGLR v. 1.0.5 (Pérez and de Los Campos 2014). Chain convergences were evaluated to define analysis parameters. Predictions were based on 35,000 Gibbs sampling iterations, in which 5000 were removed as burn-in, thinning of five and default hyper-parameters were used (for details see Pérez and de Los Campos 2014).

### Cross-validation, predictive ability, and significance tests

For all analyses, we fixed the testing population size at 200 individuals (i.e., validation population). Five random samples were obtained for training and testing populations (pseudo 5-fold validation), and testing populations were kept constant for all factors analyzed. Predictive ability for all scenarios was obtained by computing the Pearson correlation between predicted and adjusted phenotypes (LSMeans). Mean squared errors were obtained as the average squared difference between the predicted and adjusted phenotypes. In order to verify significance between the factors tested in each analysis, post hoc tests assuming Tukey correction (*σ* = 0.05) were performed, using functions implemented in the R package agricolae (de Mendiburu 2020). Since no evidence of population structure was observed in earlier studies, (Ferrão et al. 2018; de Bem Oliveira et al. 2019) we did not consider using any correction for it.

## Results

### Effect of marker and probe density

**Fig. 1:**
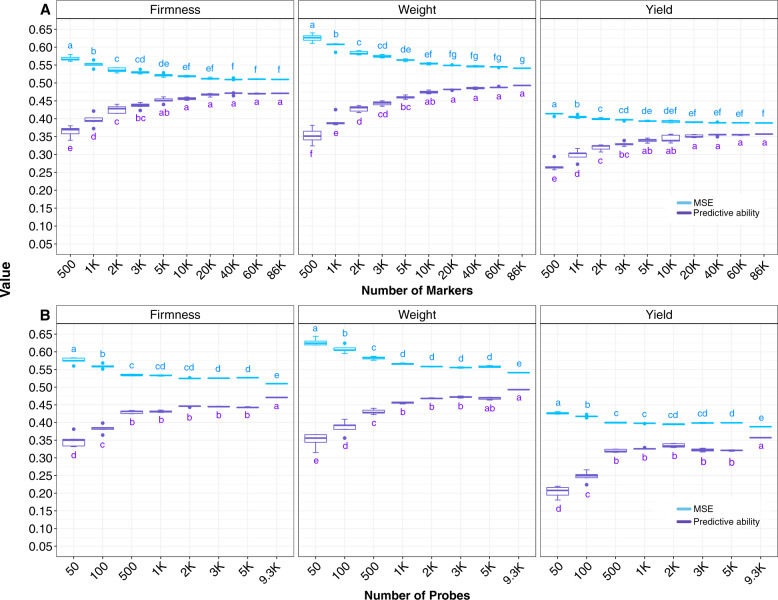
Predictive abilities and standardized mean squared error (MSE) values estimated for fruit firmness, fruit weight, and yield. Results obtained considering two scenarios: **a** under cumulative increase of markers and **b** under cumulative increase of the number of probes. Letters on top of boxplots represent the results obtained in the post hoc analysis considering Tukey correction and *σ* = 0.05, groups that share a letter are not significantly different from one another and $a > b > \ldots > z$.

Both marker density and number of probes significantly affected model performance (Fig. 1). Considering marker density, estimated values for predictive abilities varied from 0.34 to 0.47 for fruit firmness, from 0.32 to 0.49 for fruit weight, and from 0.26 to 0.36 for yield. A steep increase in predictive ability was observed for all traits when considering the interval of 500–5k markers. However, for all traits, a plateau was quickly reached, and predictive ability values obtained with 10k markers or more were not significantly different to those estimated using the full set of markers (Fig. 1a).

**Fig. 2:**
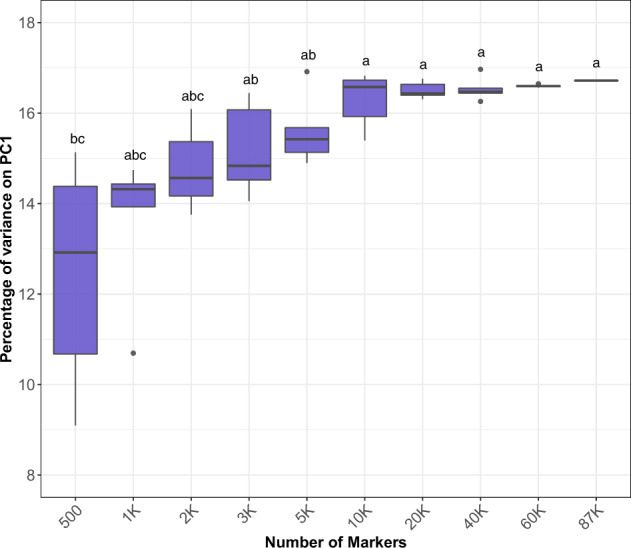
Percentages of the variance explained for the first component of the principal component analysis (PC) performed for the relationship matrices built for each marker density scenario. Letters on top of boxplots represent the results obtained in the post hoc analysis considering Tukey correction and *σ* = 0.05, groups that share a letter are not significantly different from one another and $a > b > \ldots > z$.

Similarly, the percentage of variance explained by the first principal component (PC1) obtained in the relationship matrices analyses had also reached a plateau around 10k SNPs (Fig. 2). The PC1 obtained when using <10k markers varied from 13.93 to 16.44%. When using more than 10k markers, PC1 values ranged from 16.55 to 16.97% (Fig. 2).

The probe analysis confirmed these results. In the scenario containing 50 probes, predictive ability values were as low as 0.33, 0.32, and 0.18; they reached a plateau around 0.44, 0.47, and 0.33, respectively, for fruit firmness, fruit weight, and yield when the number of probes varied between 2k and 5k. In addition, an increase of only 0.03 for predictive ability was observed when using all the 9.3k probes (Fig. 1b).

It is interesting to notice that the mean number of markers captured per probe was 18 (Fig. S1), and that with 50 probes ~550 markers were obtained, increasing when more probes were used. This is, 100 probes = ~1k markers; 500 probes = ~4.7k markers; 1k probes = ~9k markers; 2k probes = ~19k markers; 3k probes = ~28k markers; and 5k probes = ~47k markers. Therefore, when using 2k or more probes we were able to capture more than 10k markers, allowing us to generate accurate models. In addition, significantly higher bias and standard deviations were found when fewer markers and probes were used (<5k markers and <1k probes; Fig. 1).

### Effects of training population size and composition

**Fig. 3:**
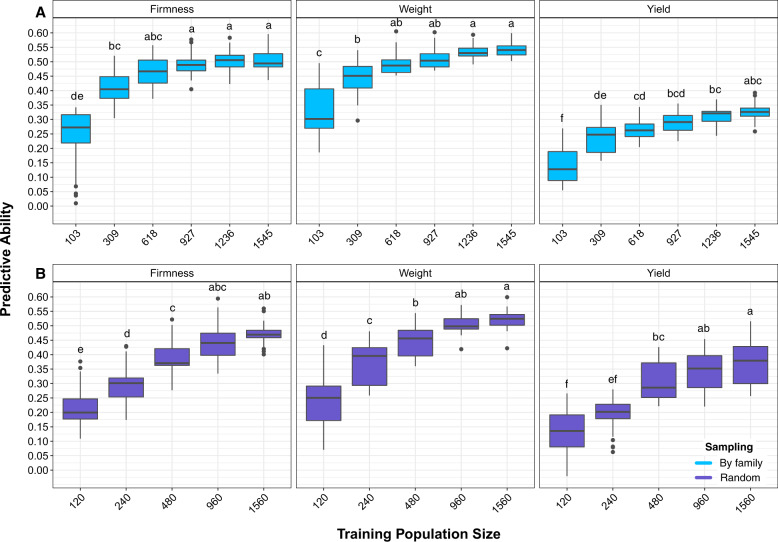
Predictive ability obtained for fruit firmness, fruit weight, and yield when considering training population size and composition. **a** Cumulative increase of the training population size considering family information and **b** cumulative increase of the training population size considering random sampling. Letters on top of boxplots represent the results obtained in the post hoc analysis considering Tukey correction and *σ* = 0.05, groups that share a letter are not significantly different from one another and $a > b > \ldots > z$. The post hoc test was performed for each trait comparing all results for both sampling scenarios.

Model performance was significantly improved with the increase of training population sizes, independent from the population composition (i.e., random sampling or using family information; Fig. 3). Significant differences between sampling methods were observed. With the exception of yield, higher predictive ability values were obtained when family information was used, even with smaller training set sizes.

For all traits, the use of ~1000 individuals has generated predictive ability values that did not differ significantly from the values obtained with the complete set of individuals (Fig. 3). In addition, when considering family information, the use of 618 individuals in the training population (6 individuals per family) resulted in predictive ability >0.46 for firmness (Fig. 3a), while similar values were only obtained for the random scenario when using all 1560 individuals for training (Fig. 3b). Along with higher predictive ability values, the use of family information has also generated more stable predictions (i.e., smaller standard deviations; Fig. S2).

### Effect of sequencing depth

**Fig. 4:**
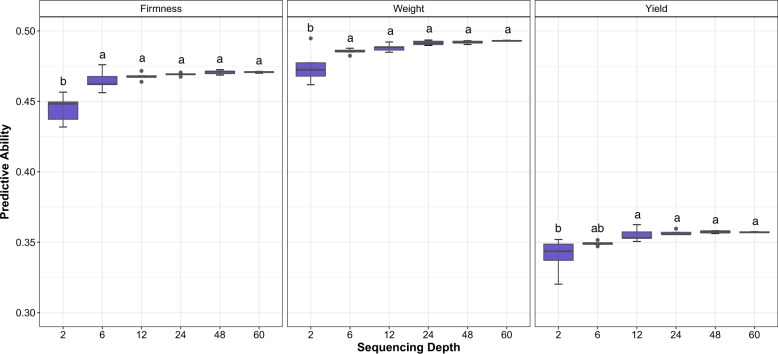
Predictive ability values obtained in genomic prediction analyses for fruit firmness, fruit weight, and yield when considering six sequencing depth scenarios. Letters on top of boxplots represent the results obtained in the post hoc analysis considering Tukey correction and *σ* = 0.05, groups that share a letter are not significantly different from one another and $a > b > \ldots > z$.

**Fig. 5:**
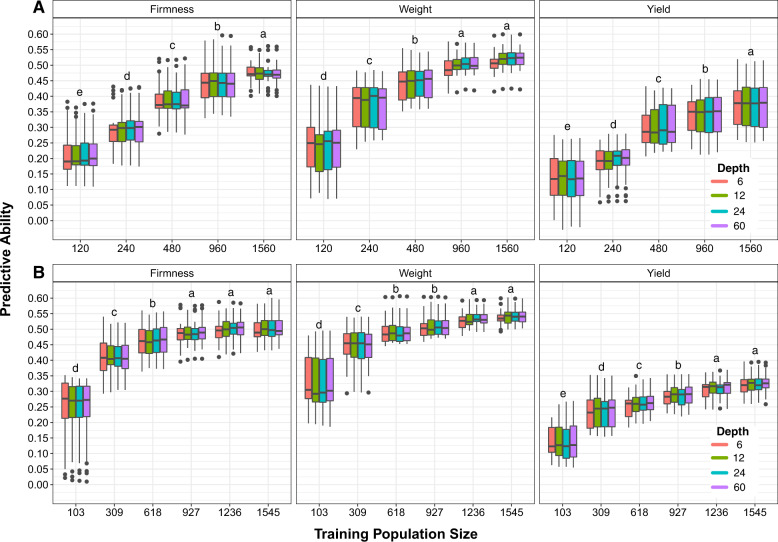
Predictive ability values obtained for fruit firmness, fruit weight, and yield when considering training population size, and sequencing depth. **a** cumulative increase of the training population size considering random sampling; and **b** cumulative increase of the training population size considering family information. Letters on top of boxplots represent the results obtained in the post hoc analysis considering Tukey correction and *σ*=0.05, groups that share a letter are not significantly different from one another and $a > b > \ldots > z$.

As the sequencing depth increased, a fast plateau of the predictive ability values was observed for all traits (Fig. 4). Sequencing depth as low as 6X yielded similar predictive ability values to those observed at higher sequencing depth scenarios, such as 60X (Fig. 4). There was no interaction between the sequencing depth used and the number of individuals in the training population, regardless of the scenario applied (i.e., random or by family; Fig. 5).

**Fig. 6:**
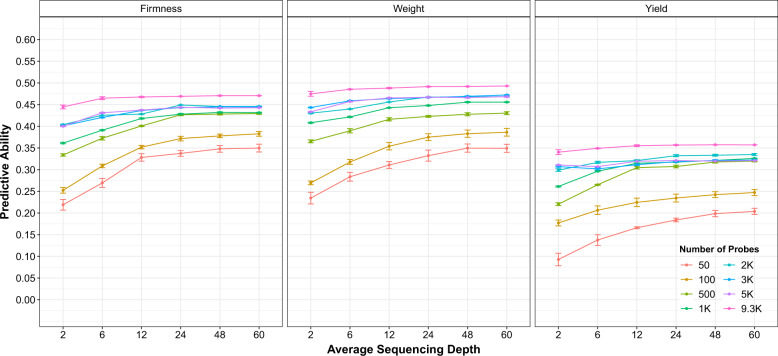
The effect of probe and sequencing depth on prediction. Predictive ability obtained for fruit firmness, fruit weight, and yield when considering probe density and average sequencing depth.

However, a significant effect of sequencing depth was observed when probe density was included; larger predictive ability values were obtained with higher sequencing depth (Fig. 6). For all traits, predictive ability plateaus with the increase of sequencing depth. This plateau was achieved faster when a higher number of probes were used. Assuming the use of at least 3k probes, sequencing depths of 12X or even 6X provided predictive ability values not significantly different from the ones obtained with higher sequencing depths (Fig. 6 and Table S2).

## Discussion

Genomic prediction has revolutionized both plant and animal breeding by significantly accelerating the selection process. In blueberry, an autotetraploid outcrossing species, the genomic prediction feasibility was recently proven and promising results are expected for increasing genetic gain and shortening the breeding cycle (de Bem Oliveira et al. 2019). In order to develop strategies to decrease sequencing costs, which limits genomic prediction implementation as a breeding tool for many species, here we evaluated the effect of training population sizes and composition, marker density, and sequencing depth on phenotype prediction. Using blueberry as a model, we show that all these factors can be substantially reduced, without significantly affecting prediction. The average predictive ability values obtained with our optimized models were 0.42, 0.45, and 0.32 for fruit firmness, fruit weight, and fruit yield, respectively. These values are moderate to high, and equivalent to the values obtained in our previous studies (Amadeu et al. 2019; de Bem Oliveira et al. 2019).

### Marker density

Genomic prediction implementation relies on high-throughput genotyping of large breeding populations. Determining a balance between predictive performance and marker density is considered a relevant outcome for practical purposes. We evaluated the impact of probes and marker densities on the predictive ability of three important traits. Notably, we observed a plateau for predictive ability for all traits when increasing marker/probe densities, illustrating that we can significantly reduce marker/probe density without negatively affecting the predictive ability. As previously described (Daetwyler et al. 2008; Wray et al. 2013, 2019), the expectation of the prediction accuracy is associated with the independent markers in which the effects can be estimated (*M*), the sample size (*N*), and the proportion of the heritability explained by the markers used ($h_M^2$, i.e., “marker heritability”). Hence, the proportion of the variance explained by the markers determines the upper limit of capturing causal effects. This factor is conditioned by the size of linkage disequilibrium blocks, since it ultimately delimits the number of independent markers that can be sampled. Thus, the increase of accuracy associated with variations in marker/probe densities can plateau, as verified in our analyses (Fig. 1). In our population, we suggest that the effective number of markers was obtained for all traits when using around 10k randomly distributed markers or when 2k probes or more were used, generating similar predictive ability results as those obtained when using the full dataset (i.e., 86,930 markers or 9390 probes). From a practical standpoint, this would represent a reduction of ~90% on marker and probe densities, which should significantly decrease sequencing cost, positively affecting the implementation of genomic prediction.

The optimization of marker density has also been reported in other crops, such as rice (Spindel et al. 2015), barley (Abed et al. 2018), and wheat (Arruda et al. 2015; Cericola et al. 2017), suggesting the use of 7k, 2k, 1.5k, and 1k markers, respectively, to obtain model performance equivalent to models using whole datasets. When compared with these previous studies, our results show that for blueberry a slightly higher number of markers would be required to maximize predictive ability and reduce costs (i.e., 10k SNPs). However, blueberry is an outcrossing, polyploid species with high heterozygosity and fast linkage disequilibrium decay (Ferrão et al. 2018; de Bem Oliveira et al. 2019). These conditions are normally related with a necessity of higher number of markers to succeed in association studies.

### Training population size and composition

The training population size used to build the prediction models has a direct effect on the cost of genomic prediction implementation, since it defines how many individuals should be genotyped and phenotyped in order to generate accurate models. Here, we investigated the effects of training population size and population composition on model accuracy, with the goal of minimizing costs. Our results indicated that predictive ability increases as training population size increases. However, as observed in other studies (e.g., Cericola et al. 2017; Norman et al. 2018), this increment was not linear and a plateau was reached (Fig. 3). Our results suggest a training population size of ~1k to achieve accurate prediction, which represents a reduction of 20% in the number of individuals to be evaluated. The decrease in training population size would contribute not only to reducing sequencing costs, but could drastically reduce the time, work, and costs involved in phenotyping and maintenance of plants in the field.

As expected, we also observed that the composition of the training population significantly affected predictive ability. Our results indicate that the use of a smaller and more representative training population, could generate a higher accuracy when compared to models built using a larger population of randomly chosen individuals. In addition, higher variation in predictive ability was observed in smaller training populations, or when family information was not considered (Fig. 3). High variance in predictive ability can impact prediction, and consequently, the long-term response to selection, which is a non-desirable risk in breeding programs (Hickey et al. 2014; Gorjanc et al. 2015).

Our results were in accordance with Hickey et al. (2014), who shows that when the relationship between the training population and the testing/selection population decreases, a higher number of individuals are necessary to achieve the same predictive performance. Relatedness is known to affect accuracies (Habier et al. 2007, 2010; Daetwyler et al. 2013; Wientjes et al. 2013). This effect is associated with the shared linkage disequilibrium blocks and its influence on the estimation of effects for each marker. Besides the linkage disequilibrium associated with physical linkage, closely related individuals are more likely to share specific causal polymorphisms and other genetic interaction effects (spurious LD), since they share a higher fraction of the genome than distantly related individuals (Lorenz and Smith 2015).

Overall, three points should be considered in genomic prediction models: population structure, relationship (family effect), and Mendelian sampling (within family effect). The use of pedigree and genomic information can be used to estimate population structure. Yet, the increase in the number of individuals for a given family can help to estimate the Mendelian sampling effect (Hickey et al. 2014). The use of family information helps to model both linkage disequilibrium and cosegregation, which can improve predictive ability and may avoid the decline in model accuracy over time (Habier et al. 2013). Therefore, increasing training population size and considering family information helped to improve model performance. That is, by capturing the effects of different genetic blocks (Mendelian sampling) in the phenotype expression, we improved the estimation of effects and consequently, improved prediction ability.

### Sequencing depth

Optimizing sequencing depth could have a major impact on genotyping costs when using a next generation sequencing platform. This is because less sequencing will be allocated per individual, enabling more samples to be multiplexed per sequencing lane (Gorjanc et al. 2015; Abed et al. 2018). Here we evaluated the effect of six depth scenarios (i.e., 2X, 6X, 12X, 24X, 48X, and 60X) on phenotype prediction.

The complexity of defining thresholds for sequencing depth in polyploids is associated with difficulty in estimating allele dosage. Given the high number of genotypic classes that these species can present, the expected signal distribution obtained during sequencing for each genotypic class progressively approximates a continuous distribution (Grandke et al. 2016; de Bem Oliveira et al. 2019). The addition of a low depth scenario in this context could increase the challenges in attributing genotypic classes. The problem here is that the misclassification of genotypes can ultimately generate bias in association analyses, resulting in an incorrect estimation for the allele effects (Grandke et al. 2016), and hampering the application of genome-based breeding for polyploids.

Uitdewilligen et al. (2013) and Bastien et al. (2018) suggest using sequencing depths of 50X–80X for an accurate assessment of allele dosage in autotetraploids. However, more modest values are shown by Griffin et al. (2011) and Gerard et al. (2018) (15X and 25X, respectively). Nevertheless, Grandke et al. (2016) shows that with next generation sequencing, no method works properly to determine allele dosage in autopolyploids. In fact, the sequencing depth adopted in polyploid studies is variable (e.g., Ashraf et al. 2014; Norman et al. 2018). To our knowledge, the impact of sequencing depth on genomic prediction for autotetraploids has not yet been addressed. Herein, by taking advantage of a large population size sequenced using high sequencing depth, we demonstrated that the depth values recommended for autotetraploid sequencing (~60X) are conservative in the genomic prediction context. We found that values as low as 6X could generate accurate predictions (Fig. 4).

The use of continuous genotypes instead of dosage parameterization could have contributed to the achievement of accurate prediction under low depth scenarios. By doing so, we avoided the bias associated with the misclassification of genotypic classes (Clark et al. 2019; de Bem Oliveira et al. 2019). However, further investigation is needed comparing ploidy standardizations (i.e., allele dosage) and continuous genotypes in the context of sequencing depth to confirm this hypothesis. Further studies could also evaluate the use of corrections for the relationship matrices, such as done by Cericola et al. (2018) or by Dodds et al. (2015). This could improve the predictive ability under lower sequencing depth scenarios, allowing the use of a very low coverage for polyploid models, such as the values obtained for livestock by Gorjanc et al. (2015).

Sequencing depth had a higher effect on predictive ability when we evaluated the number of probes/markers (Fig. 6). We show that for accurate prediction in blueberry ~20k markers (i.e., 2k probes or more) with an average sequencing depth of 12X will be needed. These results are in agreement with the Gorjanc et al. (2015) study on diploids, where for a low sequencing depth a higher number of markers were necessary to obtain the same predictive ability of models using higher depth values. Our optimized scenario would represent a decrease in marker density of 78% and a decrease of 80% on sequencing depth, when compared with the full dataset used in this study. Thus, even though a higher number of markers would be necessary to obtain accurate models (i.e., from the previously indicated 10k–20k), the allocation of resources would still be significantly affected by the reduction of sequencing depth.

For an example of the effect that changing sequencing depth could have in the allocation of resources, consider this scenario: blueberry possesses a genome of 0.6 Gb, therefore 7.2 Gb of sequencing data would be necessary to theoretically cover the genome of one sample considering a depth of 12X, while 36 Gb of data per sample would be necessary to obtain a depth of 60X. Next generation sequencing platforms available on the market, such as Illumina^®^ NovaSeq (S4 2×150), can generate up to 3 000 Gb of sequencing data when running a full flow cell. Therefore, to obtain a depth of 12X a total of 417 samples could be multiplexed per run, while to obtain a depth of 60X only 83 samples could be multiplexed per run (based on personal communication from University of Florida ICBR—NextGen DNA Sequencing).

## Conclusion

By investigating multiple combinations of genotype and phenotype scenarios, here we provide guidelines for optimizing genomic prediction implementation for blueberry breeding. We show that accurate predictions can be obtained with moderate marker density (10k, representing an eightfold decrease compared to our original dataset) and low-to-mid sequencing depth (6X–12X). Moreover, we showed that total costs for genomic prediction implementation can be significantly reduced, making use of a smaller training population size for building the prediction models (i.e., ~1k individuals), and that the use of family information to compose the training set can improve the results obtained. Altogether, our findings have important cost implications for a practical implementation of genomic prediction. The effect of this parameter reduction should be validated in future studies. While this study focused on the genomic prediction implementation for a specific breeding scheme in blueberry, the pipeline explained here can be used to improve and guide resource allocation decisions for other crops, especially polyploids.

## Supplementary information

Supplemental Figures

Supplemental Table

## Data Availability

Genotype information, as well as Supplementary information, are available at the Dryad Digital Repository: 10.5061/dryad.8pk0p2nk9. Files contained on these links are LSMeans for the phenotypes of 1804 individuals, genotype information containing information of alternative (AO) and reference alleles (RO) for 87,628 markers, and Supplementary information 1–3, which includes respectively: Supplementary figures, including Fig. S1 displaying the absolute frequency distribution for the number of markers and marker distribution considering chromosome information; Fig. S2 showing the standardized mean squared error distribution considering training population size and composition; and Fig. S3 comprising the sequencing depth distribution of the data. Supplementary tables comprising Table S1 containing the chromosome names, size, and the number of markers and probes per chromosome; and Table S2, containing the predictive ability and Tukey groups for the analysis involving the interaction between the number of probes and the sequencing depth. The authors affirm that all data necessary for confirming the conclusions of the article are present within the article, figures, and tables.
