# Supplementary material for: Optimizing whole-genomic prediction for autotetraploid blueberry breeding
Source: Heredity (Edinb). 2020 Oct 19;125(6):437–48. doi: 10.1038/s41437-020-00357-x (PMC7784927; doi:10.1038/s41437-020-00357-x)
Supplement: Supplementary file 1 — Supplemental Figures [file 41437_2020_357_MOESM1_ESM.pdf]

Supplementary Information

Optimizing whole-genomic prediction for autotetraploid blueberry breeding

Ivone de Bem Oliveira, Rodrigo Rampazo Amadeu, Luis Felipe Ventorim Ferrão,

and Patricio R. Muñoz

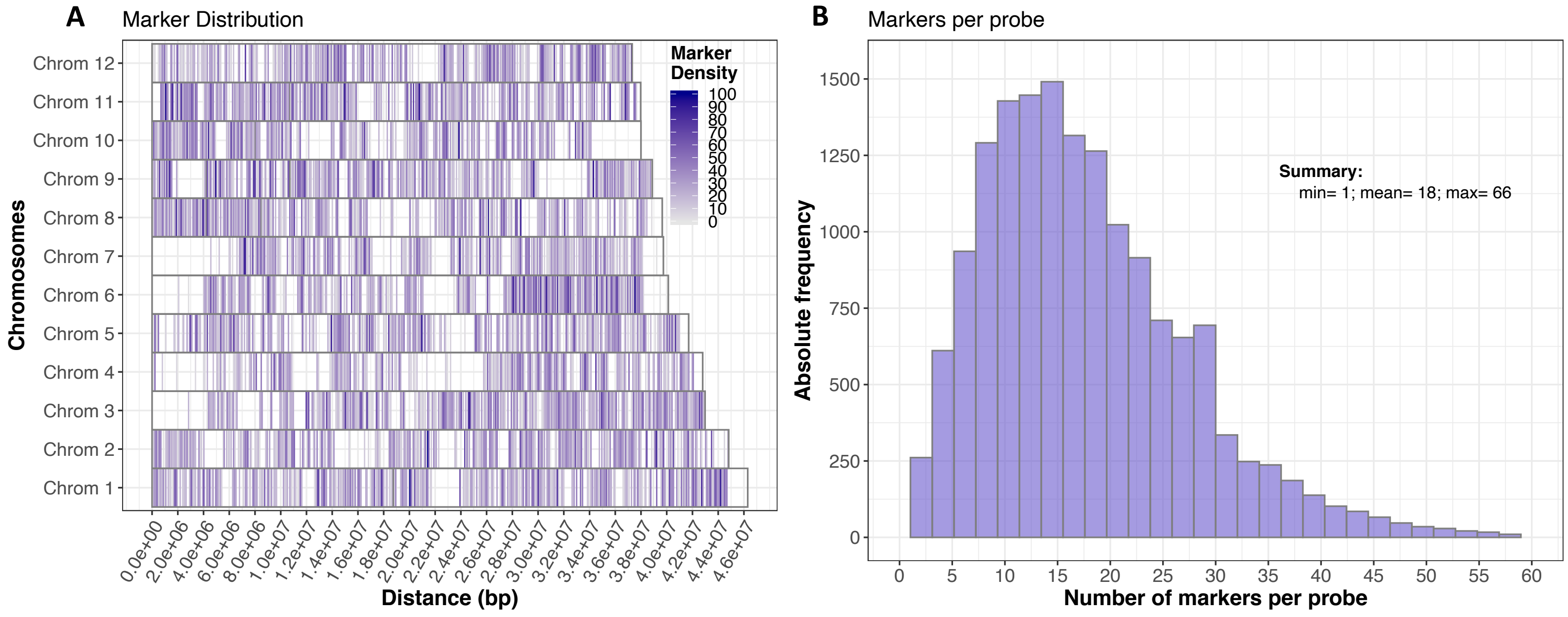

Figure S1. Marker distribution considering chromosome information **(A)**; Absolute frequency distribution for the number of markers captured by probe **(B)**.

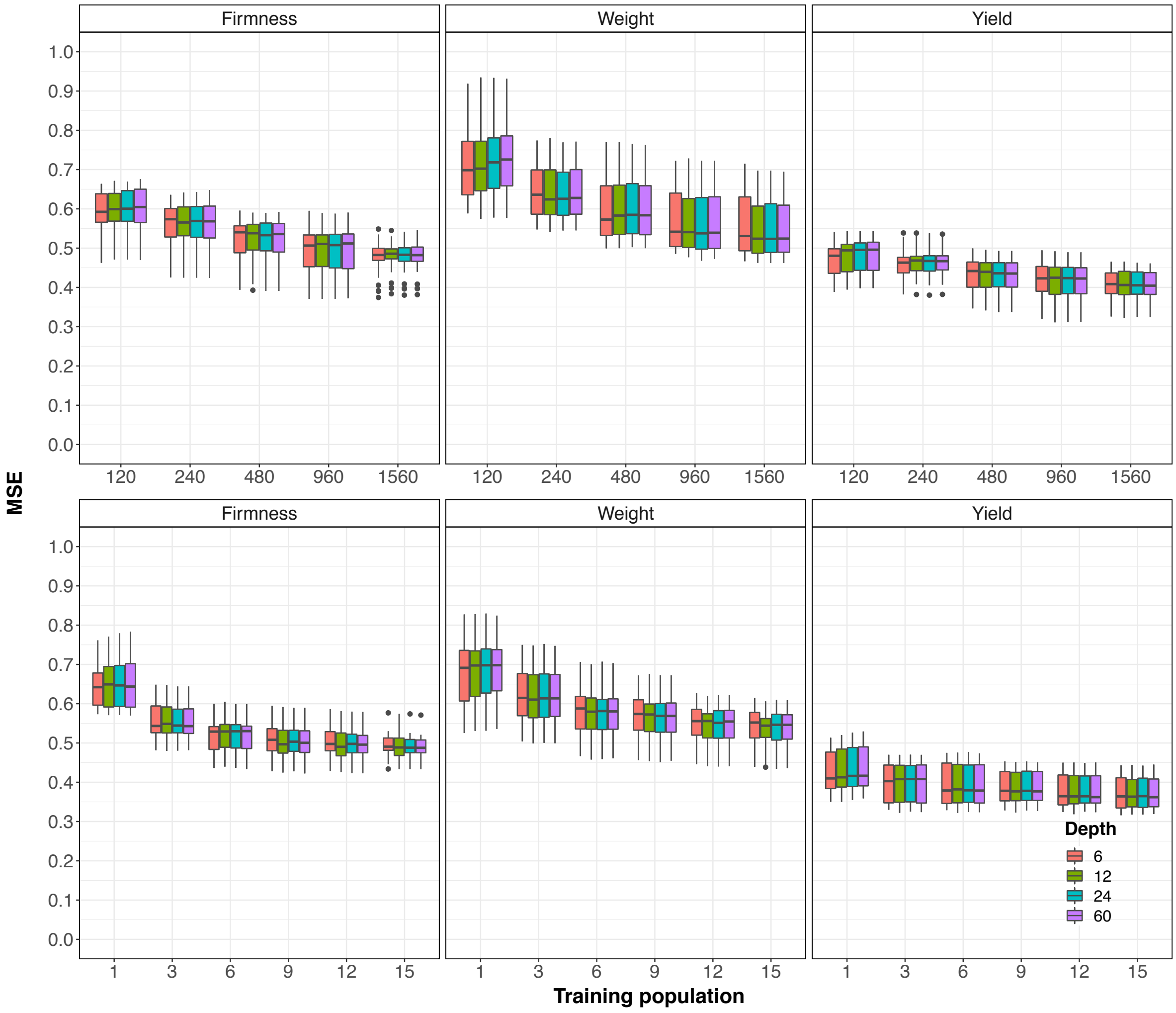

Figure S2. Standardized mean squared error obtained for fruit firmness, fruit weight, and yield when considering training population size, composition, and sequencing depth for two scenarios: (A) Cumulative increase of the training population size under random sampling; and (B) Cumulative increase of the training population considering family information

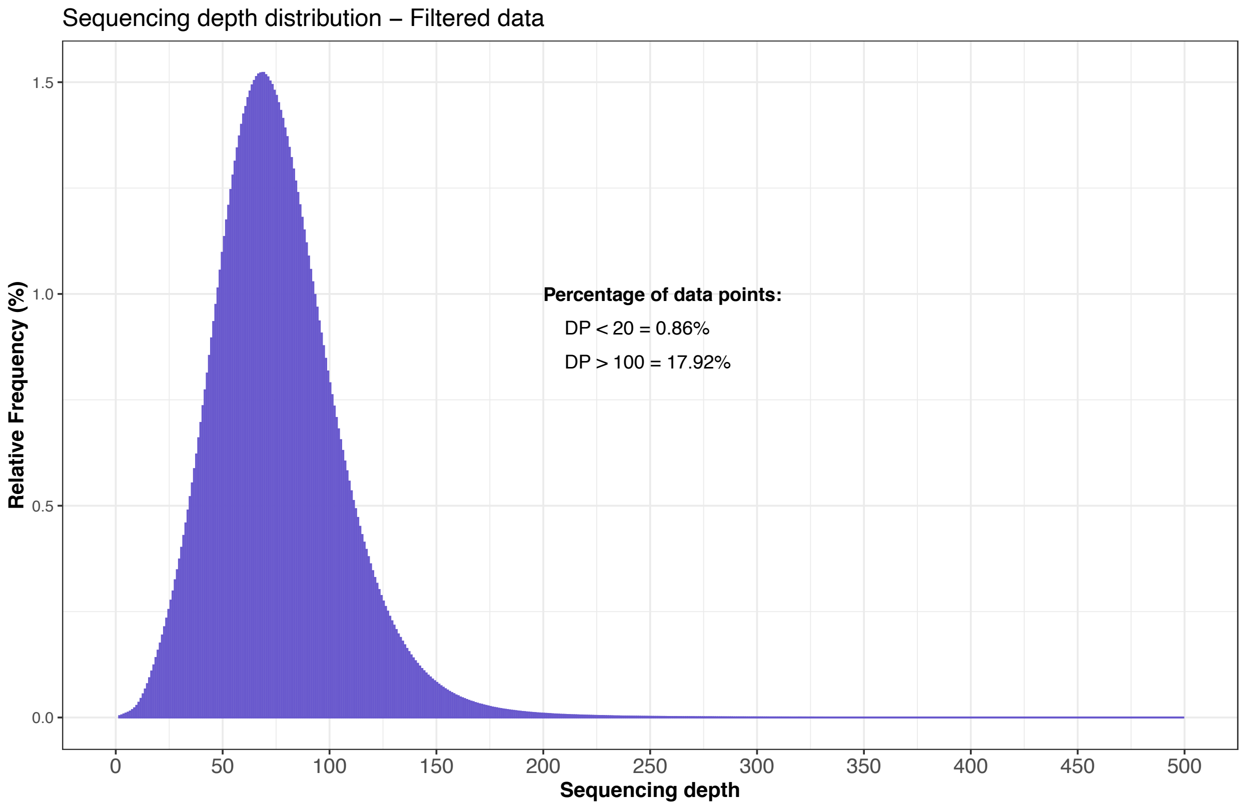

Figure S3. Sequencing depth relative distribution of 86K markers obtained for the University of Florida Blueberry Breeding population comprising 1804 individuals
