# Supplementary material for: Optimizing whole-genomic prediction for autotetraploid blueberry breeding
Source: Heredity (Edinb). 2020 Oct 19;125(6):437–48. doi: 10.1038/s41437-020-00357-x (PMC7784927; doi:10.1038/s41437-020-00357-x)
Supplement: Supplementary file 2 — Supplemental Table [file 41437_2020_357_MOESM2_ESM.pdf]

Table S1. Number of markers and number of probes per chromosome

Scaffold	Scaffold Length (Kb)	Chromosome number	Number of markers	Number of Probes
VaccDscaff1	46 295.995	1	8 793	954
VaccDscaff2	44 818.276	2	7 430	815
VaccDscaff4	42 981.373	3	8 216	892
VaccDscaff6	42 795.824	4	5 311	583
VaccDscaff7	41 705.179	5	7 163	737
VaccDscaff11	40 122.599	6	7 319	825
VaccDscaff12	39 741.682	7	5 665	590
VaccDscaff13	39 652.356	8	7 090	771
VaccDscaff17	38 874.919	9	7 824	855
VaccDscaff20	37 996.905	10	6 437	690
VaccDscaff21	37 975.728	11	8 565	904
VaccDscaff22	37 315.645	12	7 117	774
**Total**	**49 0276.481**	**12**	**86 930**	**9 390**

Table S2. Predictive ability (PA), standard deviation (SD), standard error of the mean (SE), confidence interval (95%; CI), and Tukey groups obtained for the analyses involving the number of probes and sequencing depth for fruit firmness, fruit weight, and yield.

Sequencing Depth	Probe Filter	Trait	PA	SD	SE	CI	*TukeyGroup
24	9630	Firmness	0.469	0.001	0	0.001	a
48	9630	Firmness	0.471	0.001	0.001	0.002	a
60	9630	Firmness	0.471	0	0	0.001	a
12	9630	Firmness	0.468	0.003	0.001	0.003	ba
6	9630	Firmness	0.465	0.007	0.003	0.009	cba
24	2000	Firmness	0.449	0.001	0	0.001	dcba
2	9630	Firmness	0.445	0.01	0.004	0.012	edc
24	5000	Firmness	0.444	0.001	0	0.001	edc
48	3000	Firmness	0.445	0.001	0	0.001	edc
60	3000	Firmness	0.445	0.001	0	0.001	edc
48	2000	Firmness	0.446	0.001	0	0.001	edcb
60	2000	Firmness	0.446	0.002	0.001	0.002	edcb
48	5000	Firmness	0.442	0.001	0.001	0.002	fed
24	3000	Firmness	0.443	0.001	0	0.001	fedc
60	5000	Firmness	0.443	0.001	0.001	0.001	fedc
6	5000	Firmness	0.431	0.003	0.001	0.003	gfed
12	2000	Firmness	0.428	0.002	0.001	0.002	gfed
12	3000	Firmness	0.436	0.001	0	0.001	gfed
12	5000	Firmness	0.437	0.003	0.001	0.003	gfed
24	500	Firmness	0.427	0.005	0.002	0.006	gfed
24	1000	Firmness	0.428	0.004	0.002	0.005	gfed
48	500	Firmness	0.428	0.004	0.002	0.005	gfed
48	1000	Firmness	0.432	0.003	0.001	0.004	gfed
60	500	Firmness	0.429	0.004	0.002	0.005	gfed
60	1000	Firmness	0.431	0.003	0.001	0.003	gfed
6	2000	Firmness	0.425	0	0	0.001	hgfe
12	1000	Firmness	0.418	0.003	0.001	0.004	ihg
6	3000	Firmness	0.42	0.002	0.001	0.003	ihgf
2	3000	Firmness	0.401	0.002	0.001	0.002	ji
12	500	Firmness	0.401	0.003	0.001	0.004	ji
2	2000	Firmness	0.404	0.003	0.001	0.003	jih
2	5000	Firmness	0.4	0.002	0.001	0.003	kji
6	1000	Firmness	0.391	0.003	0.001	0.004	lkj
48	100	Firmness	0.378	0.009	0.004	0.011	mlk
60	100	Firmness	0.383	0.012	0.005	0.015	mlkj
6	500	Firmness	0.372	0.009	0.004	0.011	nml
24	100	Firmness	0.372	0.012	0.005	0.015	onml
2	1000	Firmness	0.361	0.004	0.002	0.005	ponm
12	100	Firmness	0.352	0.009	0.004	0.011	qpon
2	500	Firmness	0.334	0.006	0.003	0.008	rq

24	50	Firmness	0.338	0.015	0.007	0.019	rq
48	50	Firmness	0.348	0.017	0.008	0.022	rqp
60	50	Firmness	0.35	0.02	0.009	0.025	rqpo
6	100	Firmness	0.309	0.009	0.004	0.011	s
12	50	Firmness	0.328	0.019	0.009	0.024	sr
2	100	Firmness	0.252	0.015	0.007	0.018	t
6	50	Firmness	0.269	0.023	0.01	0.028	t
2	50	Firmness	0.219	0.028	0.012	0.034	u
60	9630	Weight	0.493	0	0	0	a
12	9630	Weight	0.488	0.003	0.001	0.003	ba
24	9630	Weight	0.492	0.002	0.001	0.002	ba
48	9630	Weight	0.492	0.001	0	0.001	ba
6	9630	Weight	0.485	0.002	0.001	0.002	cba
2	9630	Weight	0.475	0.013	0.006	0.016	dcba
60	3000	Weight	0.472	0.002	0.001	0.003	dcba
48	3000	Weight	0.469	0.002	0.001	0.003	edcba
60	2000	Weight	0.469	0.001	0	0.001	edcba
60	5000	Weight	0.468	0.003	0.002	0.004	edcba
12	3000	Weight	0.464	0.003	0.001	0.003	fedcb
12	5000	Weight	0.465	0.003	0.001	0.003	fedcba
24	2000	Weight	0.468	0.002	0.001	0.002	fedcba
24	3000	Weight	0.466	0.002	0.001	0.002	fedcba
24	5000	Weight	0.467	0.003	0.001	0.004	fedcba
48	2000	Weight	0.468	0.001	0	0.001	fedcba
48	5000	Weight	0.466	0.003	0.001	0.004	fedcba
6	3000	Weight	0.459	0.002	0.001	0.003	gfedc
6	5000	Weight	0.457	0.003	0.001	0.003	hgfed
12	2000	Weight	0.456	0.001	0	0.001	hgfed
48	1000	Weight	0.456	0.002	0.001	0.003	ihgfed
60	1000	Weight	0.456	0.002	0.001	0.003	ihgfed
24	1000	Weight	0.448	0.002	0.001	0.003	jihgfed
6	2000	Weight	0.44	0.002	0.001	0.002	kjihgf
2	3000	Weight	0.443	0.002	0.001	0.002	kjihgfe
12	1000	Weight	0.443	0.001	0.001	0.002	kjihgfe
6	1000	Weight	0.422	0.003	0.001	0.003	lkj
24	500	Weight	0.423	0.005	0.002	0.006	lkj
48	500	Weight	0.428	0.008	0.004	0.01	lkji
2	2000	Weight	0.43	0.003	0.001	0.004	lkjih
60	500	Weight	0.43	0.007	0.003	0.009	lkjih
2	5000	Weight	0.433	0.003	0.001	0.003	lkjihg
12	500	Weight	0.416	0.008	0.004	0.01	mlk
2	1000	Weight	0.408	0.002	0.001	0.003	nml
48	100	Weight	0.383	0.019	0.008	0.023	on
60	100	Weight	0.386	0.02	0.009	0.024	on
6	500	Weight	0.39	0.011	0.005	0.013	onm

2	500	Weight	0.365	0.007	0.003	0.009	po
24	100	Weight	0.375	0.018	0.008	0.022	po
12	100	Weight	0.354	0.019	0.008	0.023	qp
48	50	Weight	0.35	0.021	0.009	0.026	qp
60	50	Weight	0.349	0.021	0.009	0.026	qp
6	100	Weight	0.318	0.013	0.006	0.016	r
24	50	Weight	0.332	0.028	0.013	0.035	rq
12	50	Weight	0.311	0.018	0.008	0.022	sr
2	100	Weight	0.269	0.01	0.004	0.012	t
6	50	Weight	0.284	0.022	0.01	0.028	ts
2	50	Weight	0.234	0.03	0.013	0.037	u
12	9630	Yield	0.355	0.005	0.002	0.006	a
24	9630	Yield	0.357	0.002	0.001	0.002	a
48	9630	Yield	0.357	0.001	0	0.001	a
60	9630	Yield	0.357	0	0	0	a
6	9630	Yield	0.349	0.002	0.001	0.002	ba
2	9630	Yield	0.34	0.013	0.006	0.016	cba
60	2000	Yield	0.335	0.005	0.002	0.007	dcba
24	2000	Yield	0.332	0.006	0.003	0.007	edcba
48	2000	Yield	0.333	0.005	0.002	0.006	edcba
60	1000	Yield	0.326	0.002	0.001	0.002	fedcb
2	2000	Yield	0.299	0.007	0.003	0.008	g
6	1000	Yield	0.296	0.002	0.001	0.002	g
6	3000	Yield	0.302	0.006	0.002	0.007	gf
12	500	Yield	0.305	0.007	0.003	0.009	gf
2	3000	Yield	0.307	0.003	0.001	0.004	gfe
6	5000	Yield	0.307	0.001	0.001	0.002	gfe
24	500	Yield	0.307	0.007	0.003	0.009	gfe
2	5000	Yield	0.311	0.001	0.001	0.002	gfed
12	3000	Yield	0.311	0.004	0.002	0.006	gfed
6	2000	Yield	0.317	0.006	0.003	0.007	gfedc
12	1000	Yield	0.315	0.002	0.001	0.002	gfedc
12	2000	Yield	0.321	0.004	0.002	0.005	gfedc
12	5000	Yield	0.319	0.001	0.001	0.001	gfedc
24	1000	Yield	0.317	0.002	0.001	0.003	gfedc
24	3000	Yield	0.319	0.003	0.001	0.004	gfedc
24	5000	Yield	0.321	0.001	0.001	0.002	gfedc
48	500	Yield	0.318	0.005	0.002	0.007	gfedc
48	1000	Yield	0.321	0.002	0.001	0.002	gfedc
48	3000	Yield	0.321	0.004	0.002	0.005	gfedc
48	5000	Yield	0.32	0.002	0.001	0.002	gfedc
60	500	Yield	0.32	0.004	0.002	0.005	gfedc
60	3000	Yield	0.322	0.004	0.002	0.005	gfedc
60	5000	Yield	0.321	0.001	0.001	0.002	gfedc
2	1000	Yield	0.261	0.003	0.001	0.004	h

6	500	Yield	0.265	0.002	0.001	0.003	h
60	100	Yield	0.247	0.015	0.007	0.019	ih
24	100	Yield	0.235	0.02	0.009	0.025	ji
48	100	Yield	0.242	0.015	0.007	0.018	jih
2	500	Yield	0.22	0.007	0.003	0.008	kj
12	100	Yield	0.225	0.022	0.01	0.027	kji
6	100	Yield	0.207	0.022	0.01	0.027	lk
48	50	Yield	0.199	0.016	0.007	0.02	mlk
60	50	Yield	0.204	0.016	0.007	0.02	mlk
12	50	Yield	0.166	0.005	0.002	0.006	n
2	100	Yield	0.177	0.015	0.007	0.019	nm
24	50	Yield	0.184	0.009	0.004	0.012	nml
6	50	Yield	0.138	0.028	0.012	0.034	o
2	50	Yield	0.093	0.032	0.014	0.04	p

Tukey groups obtained for the analyses involving the number of probes and sequencing depth for fruit firmness, fruit weight, and yield considering $\alpha=0.05$, groups that share a letter are not significantly different from one another and a > b > … > z
